# Prevalence of Abnormal Anal Cytology in Women with Abnormal Cervical Cytology

**DOI:** 10.31557/APJCP.2021.22.7.2165

**Published:** 2021-07

**Authors:** Perapong Inthasorn, Nuthchamon Wetpithayakom, Somsak Laiwejpithaya, Varut Lohsiriwat, Atthapon Jaishuen, Boonlert Viriyapak

**Affiliations:** 1 *Department of Obstetrics and Gynaecology, Faculty of Medicine Siriraj Hospital Mahidol University, Bangkok, Thailand. *; 2 *Obstetrics and Gynaecology Unit, Ratchaburi Hospital, Ratchaburi, Thailand. *; 3 *Department of Surgery, Faculty of Medicine Siriraj Hospital Mahidol University, Bangkok, Thailand. *

**Keywords:** Anal Cytology, anal intraepithelial neoplasia, cervical cancer, cervical cytology, human papillomavirus

## Abstract

**Objective:**

The aim of this study was to evaluate the prevalence of abnormal anal cytology in women presenting with abnormal cervical cytology (intraepithelial lesion or cervical cancer) at the largest tertiary university hospital in Thailand.

**Methods:**

A cross-sectional prospective study design was used. Anal cytology was performed on 145 women with abnormal cervical cytology between June 2014-Octoble 2014. If abnormal anal cytology was detected, anoscopy was performed with biopsy in any suspicious area of precancerous change.

**Results:**

Prevalence of abnormal anal cytology was 5.5% (8 patients). Of 8 patients, six patients presented with low-grade squamous intraepithelial lesion, one patient with high-grade squamous intraepithelial lesion, and one with atypical squamous cell cannot exclude high-grade squamous intraepithelial lesion. Abnormal anoscopic impression was found in 3 cases, as follow: The first case showed faint acetowhite lesion and anoscopic impression was low grade squamous intraepithelial lesion; the second case was reported as human papillomavirus (HPV) change by anoscopic impression; and the third case showed dense acetowhite lesion with multiple punctation and pathologic examination showed anal intraepithelial neoplasm III (AIN3). The last patient underwent wide local excision of AIN3 with split-thickness skin graft reconstruction. Final pathology confirmed AIN3 with free resection margin.

**Conclusion:**

Prevalence of abnormal anal cytology was 5.5% in patients with abnormal cervical cytology. The prevalence might be support anal cytology screening in this group of patients.

## Introduction

Worldwide the prevalence of anal cancer is low in general population. An estimated 29,000 people were diagnosed with squamous cell carcinoma of anus every year (Clifford, 2021). However prevalence has risen in the United State, with 2019 SEER data showing an increasing rate of 2.2% each year over the last 10 years (Hopp, 2021). Siegel et al., (2012) reported that the prevalence of anal cancer in men who have sex with men or HIV patients was 35 in 100,000 patients while new cases of anal cancer in female is 2.06 in 100,000 females (Salati and Kadi, 2012). It has been recently suggested that squamous cell carcinoma (SCC) of the anus may be related to sexual transmission of the human papillomavirus (HPV) in about 80-90%. Most notably HPV types 16 and 18, which were found in approximately 50% of patients with high-grade anal intraepithelial neoplasia (Domgue et al., 2019).

HPV is primarily the leading cause of cervical cancer. Cervical cancer screening by cytology is used worldwide. The prevalence of cervical cancer has dramatically decreased after the introduction and promotion of cervical cancer screening. 

However, HPV infection can be transmitted to women through receptive anal intercourse with alternative routes of transmission being possible. It was evident that women with cervical HPV infection had >3-fold increased risk of concurrent anal infection (Hernandez et al., 2005).

Calore et al., (2011) showed the prevalence of abnormal anal cytology in women with abnormal cervical cytology about 59.2% (29/49 patients) and abnormal cervical cytology of their study was 2 atypical squamous cell of undetermined significance (ASC-US), 27 low- grade intraepithelial lesion (LSIL) and 20 high-grade squamous intraepithelial lesion (HSIL). They proposed that anal mucosa would be a reservoir of HPV. Anal cytology in women who had abnormal cervical cytology may be the early detection for the precancerous lesion of anal cancer.

Relatively little is known about the prevalence of abnormal anal cytology in healthy women with abnormal cervical cytology. Accordingly the primary objective of this study was to investigate the prevalence of abnormal anal cytology in healthy women who had abnormal cervical cytology.

## Materials and Methods

From June 2014 – October 2014, we enrolled patients with abnormal cervical cytology. Sample size was calculated by nQuery Advisor program software. Previous study reported prevalence of abnormal anal cytology in women with abnormal cervical cytology to be about 59.2% (Calore et al, 2011). With acceptable error of 8% (15% of 59.2) and 95% confidence, sample size was estimated to be 145 cases. The study was approved by the Siriraj Institutional Review Board (Si298/2014). Written informed consent was obtained from all study participants. Inclusion criteria were women aged ≥ 18 years, with abnormal cervical cytology (intraepithelial lesion or invasive cervical cancer) within 1 month before getting anal cytology test. Cervical cytology result was classified according to Bethesda 2001 system (Smith, 2002).

The subjects were excluded from this study if they were immunocompromised, had the history or presence of gastro-intestinal tract malignancy or hemorrhage or anal wound. 

*Anal specimen collection*

Anal cytology test was collected within one month after cervical cytology. Two non absorbable swabs moistened with normal saline solution were used to collect anal specimen. The swab was inserted into the anus 4 cm. beyond the external anal sphincter and rotated 360º clockwise 2 times (Chaves et al., 2012). The swab was then placed in the Siriraj Liquid Base solution of 5 ml. and prepared for Siriraj liquid based cytology technique (Laiwejpithaya et al., 2008). Anal cytology was interpreted by one of three experienced cytologists and was reviewed by consultant of gynaecologic cytology unit (S L). Specimen adequacy was based primarily upon specimen cellularity and morphological quality of the sample (Smith, 2002). In case of unsatisfactory anal cytology the procedure was repeated. Anal cytology and anal histology were reported based on a guideline proposed by Ortoski and Kell (2011). Anal cytology are reported as ASCUS, atypical squamous cell can not exclude HSIL (ASC-H), LSIL, HSIL and squamous cell carcinoma (SCC). Anal histology are reported as AIN1, AIN2, AIN3 and invasive anal carcinoma. 

*Questionnaire*

After informed consent, a self questionnaire was performed at enrolled date. A short survey was queried on demographic information. The questions also concerned the number of sexual partners, anal intercourse, sexually transmitted disease (STD) in the lifetime and HPV vaccine injection.

Treatment of abnormal cervical cytology was routinely performed. A colorectal surgeon(LW) was consulted to manage the patients with abnormal anal cytology. Magnified anoscopy was performed in all patients who had abnormal anal cytology. If the anoscopic study showed any abnormality, anal biopsy was done for histopathology. Further treatment of anal disease was managed as appropriate by colorectal surgeon. 

*Statistical Analyses*

The demographic characteristics were summarized using frequencies and percentages. Fisher’s exact were used to compared variables between normal and abnormal anal cytology. The significant level was p<0.05. Analyses were done with SPSS version 20.

## Results

A total of 145 women with abnormal cervical cytology were enrolled to this study. Age ranged from 19-84 years with median age of 39 years. Seventy three (50.7%) of the subjects had forty or less years old. Eighty six (59.3%) had first time of sexual intercourse ≤ 21 years old. Nine (6.2%) women reported anal intercourse. Six (4.1%) women had a history of at least one sexually transmitted disease. Seven (4.8%) women received HPV vaccine. Eighty five (58.6%) had more than two pregnancy. One hundred and twenty five (86.2%) had cervical cytology more severe than ASCUS. Among variable risk factors, more than two parity was the only prognostic factor for abnormal anal cytology. The demographic data were shown in Table 1.

For abnormal cervical cytological results, twenty (13.8%) women had ASC-US, 18 (12.4%) women with ASC-H, 59 (40.7%) with LSIL, 34 (23.4%) with HSIL and 5 (3.5%) with squamous cell carcinoma. Nine (6.2%) had atypical glandular cell (AGC) which were enumerated to 4 cases of endocervical cell type, 2 cases of endometrial cell type and 3 cases of not otherwise specify.

Anal cytology was performed in all women with abnormal cervical cytology. Anal cytological abnormality was found in 8 (5.5%) and classified as 1 case with anal ASC-H, 6 cases with LSIL and 1 case with HSIL. Of note, 2 patients with high-grade anal abnormality (1 HSIL and 1 ASC-H) came from high-grade cervical abnormality. Details of patients with abnormal anal cytology were shown in Table 2. 

**Figure 1:**
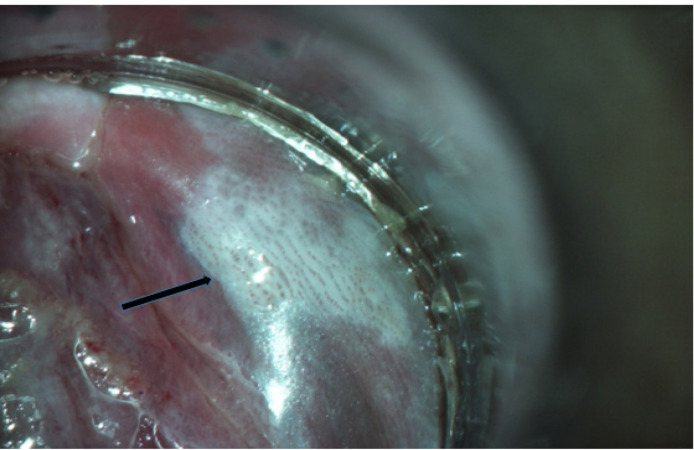
Anoscopy Showed Dense Acetowhite Lesion with Punctuation (Black Arrow)

Anoscopy was performed in all cases who had abnormal anal cytology. Table 2 showed details of the patients with abnormal anal cytology. Abnormal anoscopic finding was found in 3 cases. About the patients with abnormal anoscopic finding, one case showed faint acetowhite lesion and anoscopic impression was LSIL . One case was reported as the HPV change by anoscopic impression. One case showed dense acetowhite lesion with multiple punctuation and anoscopic impression was HSIL (Figure 1). Histologic results of the first two cases were normal anal mucosa and histologic result of the third case was AIN 3. In case of AIN 3, barium enema was performed and reported as normal study. The last patient underwent wide local excision of AIN3 with split-thickness skin graft reconstruction. Final pathology confirmed AIN3 with free resected margin. She is now alive without disease.

**Table 1:** Demographic Data and Risk Factors for Abnormal Anal Cytology

Factors	Number (%)	Normal anal Pap (%)	Abnormal anal Pap (%)	Prevalence ratio	95% confidence interval	P value
Age				0.44	0.05-3.97	0.41
≤40years	73 (50.7%)	68 (93.2%)	5 (6.8%)			
>40years	71 (49.3%)	68 (95.8%)	3 (4.2%)			
Partner				1.16	0.28-4.82	0.56
1	77 (53.5%)	73 (94.8%)	4 (5.2%)			
>1	67 (46.5%)	63 (94%)	4 (6%)			
Age of first intercourse				0.87	0.20-3.78	0.58
≤21years	86 (59.3)	81 (94.2%)	5 (5.8%)			
>21 years	59 (40.7%)	56 (94.9%)	3 (5.1%)			
History of STD				0.27	0.23-2.59	0.29
Yes	6 (4.1%)	5 (83.3%)	1 (16.7%)			
No	139 (95.9%)	132 (95%)	7 (5%)			
Anal intercourse				0.43	0.47-3.97	0.41
Yes	9 (6.2%)	8 (89.9%)	1 (11.1%)			
N0	136 (93.8%)	129 (94.9%)	7 (5.1%)			
HPV vaccine				0.32	0.03-3.04	0.33
Yes	7 (4.8%)	6 (85.7%)	1 (14.3%)			
No	138 (95.2%)	131 (94.9%)	7 (5.1%)			
Number of parity				N/A	N/A	0.01*
≤2	60 (41.4%)	60 (100%)	0 (0%)			
>2	85 (58.6%)	77 (90.6%)	8 (9.4%)			
Cervical Pap				N/A	N/A	0.29
ASCUS	20 (13.8%)	20 (100%)	0 (0%)			
>ASCUS	125 (86.2%)	117 (93.6)	8 (6.4%)			

**Table 2:** Details of Patients with Aabnormal Anal Cytology

Case	Parity	Anal Intercourse	HPV Vaccine	Cervical Cytology	Anal Cytology	Anoscopic Impression	Anal Histology
1	2	Never	Ever	LSIL	LSIL	LSIL	Normal
2	2	Never	Never	LSIL	LSIL	HPV change	Normal
3	2	Ever	Never	HSIL	HSIL	Normal	-
4	4	Never	Never	LSIL	LSIL	Normal	-
5	2	Never	Never	LSIL	LSIL	Normal	-
6	2	Never	Never	HSIL	LSIL	Normal	-
7	3	Never	Never	HSIL	ASC-H	HSIL	AIN3
8	2	Never	Never	LSIL	LSIL	Normal	-

## Discussion

High risk HPV was known as carcinogen for cervical cancer. In addition the association between anal cancer and sexually transmitted disease was found in the past. Although our study did not investigate the effect of high risk HPV and abnormal anal cytology. There was evidence that anal cancer was related with HPV infection, especially HPV type 16 and/or 18. The 2 most common high-risk HPV types were detected in 50% of high-grade anal intraepithelial neoplasia (Domgue et al., 2019).

Auto-infection would be a cause of re-infection of the cervix. Anal mucosa would be a possible reservoir of HPV in patients with cervical HPV infection (Calore et al., 2011). A step to prove this hypothesis is to study the prevalence of abnormal anal cytology in women who had abnormal cervical cytology.

Prevalence of abnormal anal cytology in HIV infection was high to 26% (Patarapadungkit et al., 2012). The abnormal anal cytology in women infected with HIV was associated with abnormal cervical cytology, CD4 < 200 cell/mm3 and smoking (Chaves et al., 2012) .

Calore et al., (2011) reported that among 49 healthy women with abnormal cervical cytology, 29 (59.2%) patients showed abnormal anal cytology. They also revealed the high prevalence (49%) of anal intercourse in the subjects. Our study reported 8 (5.5%) cases of abnormal anal cytology. Three reasons may explain the lower prevalence of our study. The first reason might be from the difference in sexual behavior compared to the previous study such as anal intercourse between two studies. Anal intercourse was found only 9 (6.2%) cases in our study.. Second, from the difference in distribution of abnormal cervical cytology, Calore et al (2011) included only 4% of ASCUS and 40% of HSIL of cervical cytology while 13.8% of ASCUS and only 23.4% of HSIL were included in our study. The last reason might be difference in a period of anal cytology specimen collections. Calore et al., (2011) included patients in a period not exceeding 1 week of abnormal cervical cytology while not exceeding 1 month in our study. Santoso et al found that patients with genital intraepithelial neoplasia have 12.2% prevalence of anal intraepithelial neoplasia. Five per cent HIV positive and 32% prevalence of anal sex were found in their study. However Koppe et al., (2011) found that the prevalence of anal intraepithelial neoplasia was 6.6% which was comparable to prevalence of our study .

Anal intercourse may increase traumatic chance of anal mucosa. HPV can easily transfer to rectal mucosal cell and might trend to develop high prevalence of abnormal anal cytology. Hosseini et al., (2018) showed that the patients who had anal intercourse showed more positive anal cytology than those who did not significantly. However some studied reported no association between anal intercourse and the presence of anal HPV infection (Law et al., 1991). In our study, case of AIN 3 had never had anal intercourse. The hypothesis of rectal mucosa reservoir from contamination of vaginal discharge may be cause of abnormal anal cytology. 

In our study, median age of the patients was 39 years old. We found that age of the patients (≤40 vs > 40). Nobre et al (2016) and Hosseini et al (2018) also found age was not the risk factor for abnormal anal cytology.

Our study revealed that only number of pregnancy (≤ 2 vs >2) was the prognostic risk factor for abnormal anal cytology (p=0.01). Study showed that high parity was associated with high risk HPV and cervical cancer (Berraho et al.,2017). Goodman et al., (2010) and Lamme et al., (2014) revealed that genital and anal HPV infection was common to occur at the same time. We could not find an association between abnormal anal cytology and other risk factors ( number of partners, age of first intercourse, history of STD, receiving HPV vaccine). This might be too small sample size to find these risk factors. However Moscicki et al., (1999) could not find any association between these risk factors and abnormal anal cytology too.

Although Hosseini et al., (2018) found HSIL cervical cytology was a risk factor for abnormal anal cytology. Anal intraepithelial neoplasia, especially high-grade, is the main precursor of anal cancer (Santoso et al., 2010). Thus, early detection and treatment of anal intraepithelial neoplasia should reduce the frankly anal cancer. However we could not find any association between severity of cervical cytology and abnormal anal cytology, Again this might be risk factors did not the primary objective of our study and we did not calculate sample size to find for risk factors. 

In conclusion, the prevalence of 5.5% of abnormal anal cytology in patients with abnormal cervical cytology may support anal cytology screening in this group of patients. However the further study may focus on the factors that could predict the high grade anal cytology. 

## Author Contribution Statement

PI was involved in study conception, data collection,analysis and manuscript preparing. NW was involved in study conception,analysis. SL was involved in reviewing cytology. VL was involved in performing anoscopy. AJ and BV were involved in data collection. All authors reviewed the results and approved the final version of the manuscript.
